# Newborn infants' auditory system is sensitive to Western music chord categories

**DOI:** 10.3389/fpsyg.2013.00492

**Published:** 2013-08-07

**Authors:** Paula Virtala, Minna Huotilainen, Eino Partanen, Vineta Fellman, Mari Tervaniemi

**Affiliations:** ^1^Cognitive Brain Research Unit, Cognitive Science, Institute of Behavioural Sciences, University of HelsinkiHelsinki, Finland; ^2^Finnish Centre of Excellence in Interdisciplinary Music Research, University of JyväskyläJyväskylä, Finland; ^3^Finnish Institute of Occupational HealthHelsinki, Finland; ^4^Children's Hospital, Helsinki University Hospital and University of HelsinkiHelsinki, Finland

**Keywords:** auditory processing, electroencephalography (EEG), event-related potentials (ERP), mismatch negativity (MMN), music, enculturation, development

## Abstract

Neural encoding of abstract rules in the audition of newborn infants has been recently demonstrated in several studies using event-related potentials (ERPs). In the present study the neural encoding of Western music chords was investigated in newborn infants. Using ERPs, we examined whether the categorizations of major vs. minor and consonance vs. dissonance are present at the level of the change-related mismatch response (MMR). Using an oddball paradigm, root minor, dissonant and inverted major chords were presented in a context of consonant root major chords. The chords were transposed to several different frequency levels, so that the deviant chords did not include a physically deviant frequency that could result in an MMR without categorization. The results show that the newborn infants were sensitive to both dissonant and minor chords but not to inverted major chords in the context of consonant root major chords. While the dissonant chords elicited a large positive MMR, the minor chords elicited a negative MMR. This indicates that the two categories were processed differently. The results suggest newborn infants are sensitive to Western music categorizations, which is consistent with the authors' previous studies in adults and school-aged children.

## Introduction

Western tonal music has two modes, major and minor. Major and minor chords differ in their interval structure, i.e., in the mutual relationships between the notes of the chord. The major and minor chords differ by one semitone (the smallest possible interval difference) in one of the three notes of a triad chord. This small physical difference causes a musically meaningful contrast, since major and minor differ in their emotional connotations (Pinchot Castner and Crowder, 1990; Hunter et al., 2010). In music, chords can be replaced by their different inversions, i.e., by shifting some notes of the chords to adjacent octaves. This retains the chords' identity as major or minor, despite changes in its physical composition. The difference between a chord and its inversion is physically larger than the difference between major and minor chords, when taking into account only the interval size. However, chords and their inversions elicit different neural processes. For example, we recently demonstrated that highly dissonant chords and minor chords were discriminated in the context of consonant root major chords, *while the inverted major chords were not* (Virtala et al., 2011, 2012). This occurred even though inverted major chords, as well, introduce a change in interval structure. These results provide evidence that the discrimination of highly dissonant and minor chords from consonant major chords cannot be due to the interval structure change itself, but due to the specific types of interval structure changes introduced in dissonant and minor chords.

In addition to major vs. minor, consonance vs. dissonance is a central dichotomy in Western tonal music. In music theory, consonance has been described as something harmonious and stable, whereas dissonance is considered unpleasant and in need of resolution (see e.g., Rossing et al., 2002). In chords, consonance vs. dissonance is defined by specific interval structures causing harmonious or unharmonious combinations. There is a continuum between consonance and dissonance, with the minor chord being somewhat more dissonant than the major chord, and dissonant chords, such as those used by Virtala et al. (2011, 2012), being much more dissonant than the major chord. There are two main theories for the perception of consonance and dissonance. According to Plomp and Levelt (1965), the sensation of dissonance arises when the fundamental frequencies or harmonic partials of two or more simultaneous tones lie within a critical band on the basilar membrane, which leads to beating or the sensation of “roughness.” According to McDermott et al. (2010), on the other hand, consonance is related to how closely all of the tones match simple harmonic proportions. For the purposes of the present investigation, the important point is that both of these theories point to a peripheral origin for consonance and dissonance. Consonant and dissonant intervals produce different firing patterns of the auditory nerve (Tramo et al., 2001) and have different neural correlates in the brainstem (Bidelman and Krishnan, 2009). Furthermore, the degree of dissonance in chords correlates with the magnitude of phase-locked oscillatory activity in the auditory cortex of humans and monkeys (Fishman et al., 2001).

**Figure 1:**
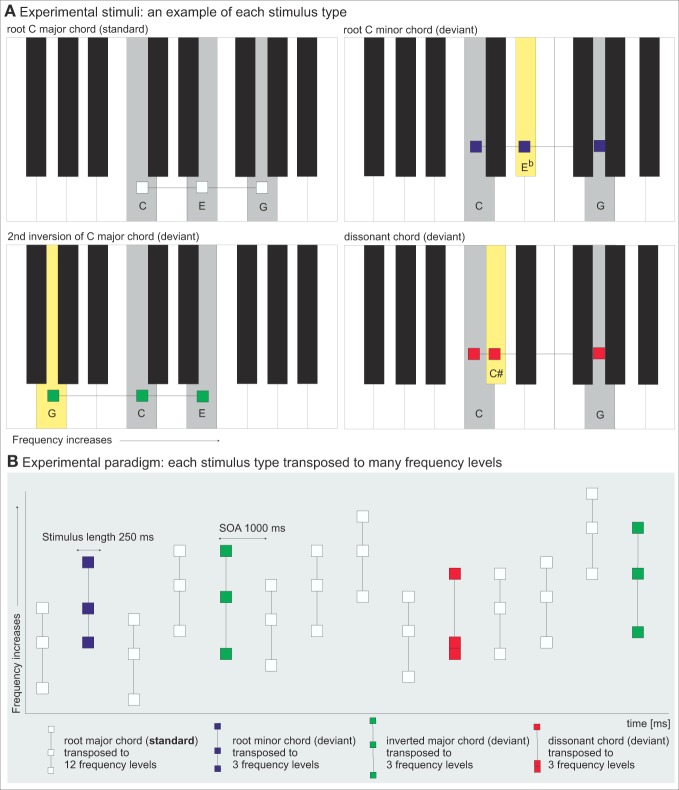
**(A)** Experimental stimuli. One example of each stimulus type illustrated with gray-shaded keys on the piano keyboard. The yellow key illustrates the deviant chords' difference from the root major chord (standard). **(B)** Experimental paradigm. The oddball sequence with root major chords (white) as standards and root minor chords (blue), inverted major chords (green) and dissonant chords (red) as deviants, each transposed to various frequency levels.

Sensitivity to consonance and dissonance has been demonstrated in young infants (Trainor et al., 2002). Furthermore, the discrimination of consonance vs. dissonance seems to be present in some other species as well (e.g., monkeys, Izumi, 2000, and birds, Hulse et al., 1995). Humans also *prefer* consonance over dissonance, and this preference seems to be culture-independent to some extent (Butler and Daston, 1968; Fritz et al., 2009). For example, a native African population naïve to Western music seemed to prefer sensory consonance in Western music (Fritz et al., 2009). On the other hand, in another study, Indian listeners judged the dissonant sounds to be less “in need of resolution” than Canadian listeners, indicating a cultural influence on the conceptions of dissonance in these groups (Maher, 1976). Some studies have shown preference of consonance over dissonance in other species (in chicks, Chiandetti and Vallortigara, 2011), while others have not (in tamarine monkeys, McDermott and Hauser, 2004). Preference is more than just discrimination, since it can be interpreted as a pleasantness rating. However, the preference of consonance is not without complexity, since mere continuous consonance is hardly considered pleasant, let alone interesting, by musicians (Rossing et al., 2002). Moreover, consonance-dissonance in music is a complex continuum rather than a simple dichotomy, and, ultimately, culture and conventions define where the line is drawn (Rossing et al., 2002). In a recent study, music training and chord familiarity also had a large effect on consonance-dissonance ratings of chords (McLachlan et al., 2013). A study of consonance judgments showed that musically trained and untrained subjects considered major chords more consonant than minor chords, and, furthermore, root chords more consonant than inverted chords (Roberts, 1986). Indeed, the combinations of the harmonic partials in the minor chords' notes introduce somewhat more dissonance than in the major chords. This is why minor chords are slightly more dissonant “by nature” than major chords. They are, however, considered consonant chords in Western music and carry a specific emotional meaning in the Western music culture. Figure 1A illustrates the interval structures in different Western music chord types: root major and minor chords and an inverted chord. These are all presently considered consonant or quite consonant chords in Western music. An example of a highly dissonant interval structure in a triad chord is also given: one semitone, small second, followed by six semitones, a so-called tritone.

The present study addressed Western music chord processing in the newborn infant by employing event-related potentials (ERPs) (Luck, 2005) in a mismatch negativity (MMN) paradigm (Näätänen et al., 2007). MMN is a change-specific ERP component reflecting central auditory processing and sound categorization. In adults, it is seen as a negative ERP displacement at frontocentral electrode locations when a deviant stimulus is presented in a context of frequently repeating standard stimuli (Näätänen et al., 2007). MMN has been used widely in studies of infants, due to the non-invasiveness of the measurement and lack of attention demands, since MMN can even be elicited while asleep (Alho et al., 1990). MMN magnitude has also been shown to be associated with response accuracy in behavioral discrimination tasks (Tiitinen et al., 1994; Amenedo and Escera, 2000; Novitski et al., 2004). In infants, change-related responses to auditory stimuli are present already at birth (e.g., Alho et al., 1990; He et al., 2007, 2009; Novitski et al., 2007) and even during the fetal period (Huotilainen et al., 2005). However, they may vary in latency and polarity compared to the adult MMN (e.g., Alho et al., 1990; Cheour-Luhtanen et al., 1995; Trainor et al., 2003; He et al., 2007, 2009). Furthermore, change-related ERPs in infants have been shown to correlate with both the gestational age and cardiac measures related to vagal tone and maturation (e.g., Porges et al., 1999; Leppänen et al., 2004). In a recent review paper, Trainor (2012) points out that due to the layer-specific pattern of cortical maturation during infancy, more positive ERP components are expected than later on. In this study, to avoid confusion, change-related enhancements in ERPs, whether positive or negative in polarity, are called mismatch responses (MMRs) and considered an infant counterpart of the adult MMN.

ERP studies have shown that the auditory system of newborn infants can discriminate sound properties like pitch, duration and location (for a review, see Trehub, 2003), as well as more complex sound features based on e.g., abstract rules that define the order of the stimuli (Ruusuvirta et al., 2003; Carral et al., 2005). Previous behavioral and EEG studies suggest that infants demonstrate the readiness to process music during the first months of life. For example, infants can learn to recognize a melody, even when it is transposed to different keys (Plantinga and Trainor, 2005, 2009; Tew et al., 2009). These results indicate that infants are able to process global, relational properties of sound stimuli, rather than only their absolute frequencies—a necessity in understanding music as well as speech. Furthermore, behavioral and electrophysiological studies have shown that infants can differentiate changes in central musical properties like tempo (Baruch and Drake, 1997) and beat (Winkler et al., 2009).

Trainor and Trehub (1992) demonstrated that when compared to adults, infants are better at discriminating sound properties independently of music culture: a change in melody that is consistent with Western music harmony is more easily discriminable for infants than for adults. The same has been reported for rhythm: while adults seem to process the rhythmic structure via the rhythmic elements typical to their own music culture, infants are more able to adapt their perception to a new rhythmic structure in music. In fact, after hearing a new rhythmic structure for a short while, infants are able to discriminate changes in it, unlike adults (Hannon and Trehub, 2005a,b). However, during the first year of life babies start to prefer the rhythmic structure of their own culture, and most likely this selective attention to familiar music enhances the culture-specific processing (Soley and Hannon, 2010). Importantly, not even newborn infants are free from cultural influences and experience-dependent learning. Simple auditory memory is functional during the fetal period (Draganova et al., 2005; Huotilainen et al., 2005). For example, melodies or characteristics of speech that the fetus has heard *in-utero* can be recognized by the infant after birth (Lecanuet and Schaal, 1996; Moon and Fifer, 2000).

To our knowledge, no studies have examined major vs. minor discrimination in infants using electrophysiological measures. Behavioral studies show no preference for major vs. minor chords in infancy (Crowder et al., 1991), but a preference for musical consonance over dissonance has been found (Zentner and Kagan, 1998; Trainor et al., 2002). The preference for consonance seems to be present at birth, and might even be independent of prenatal exposure to music, since newborn infants of deaf parents prefer consonance over dissonance as much as newborn infants of hearing parents (Masataka, 2006). Also, like adults, infants judge two musical intervals to be more similar when they are both consonant than if one is dissonant, regardless of the interval size difference (Schellenberg and Trainor, 1996). This suggests that the infant auditory nervous system is sensitive to the consonance-dissonance dichotomy of the intervals, in addition to the interval size *per se*. More recently, a functional magnetic resonance imaging (fMRI) study showed sensitivity to consonant vs. dissonant melodies in the auditory system of newborn infants (Perani et al., 2010).

In the present study, a chord paradigm developed for previous studies on adults and school-aged children was used on newborn infants. Previously we demonstrated that highly dissonant and minor chords but not inverted major chords elicited statistically significant MMNs in the context of consonant root major chords in non-musician adults (Virtala et al., 2011). In a second study, using a modified paradigm with minor and inverted major chords in the context of root major chords, minor chords elicited MMNs in musically trained 13-year-olds but not in 13-year-olds without musical training (Virtala et al., 2012). Furthermore, the inverted major chords elicited MMNs in neither of the groups of children in the study (Virtala et al., 2012). Taken together, these results suggest that general neural development or, more specifically, passive exposure to Western music during development may improve the neural discrimination of some Western music categories. This is evident because non-musician adults could neurally discriminate minor from major chords whereas 13-year-old children without musical training did not demonstrate this ability. However, formal musical training seems to enhance the attainment of these neural discrimination skills, since musically trained 13-year-olds were able to neurally discriminate minor from major chords. Since inverted major chords did not elicit statistically significant MMNs in any of the subject groups, the role of musical training and passive exposure to Western music in mastering this discrimination is left open for future studies.

The aim of the present study was to determine whether minor, inverted major and dissonant chord interval structures are neurally encoded by the infants as they are by non-musician adults (Virtala et al., 2011) and musically trained children (Virtala et al., 2012). Sensitivity to chords at birth would indicate a readiness to discriminate between Western music chord categorizations. To this end, we investigated whether minor, inverted major and dissonant chords in a context of consonant root major chords can elicit MMRs, when no novel frequencies are present in the deviant stimuli. Since earlier studies show that consonance-dissonance discrimination happens in infancy (Zentner and Kagan, 1998; Trainor et al., 2002; Perani et al., 2010), we hypothesized that this discrimination is also visible in the ERPs. We aimed to test whether newborn infants would be able to discriminate minor chords from major chords, since, in our previous study, 13-year-olds without musical training did not show MMN responses to this contrast in the “ignore” condition of our experiment (Virtala et al., 2012). Also, we hypothesized that newborn infants might even discriminate inverted major chords from root major chords. Since they only have minimal exposure to Western music where root and inverted major chords can often replace each other, newborn infants might be sensitive to the interval structure difference between root and inverted major chord.

## Methods

### Participants

The participants were healthy newborn infants (*n* = 28) born in the Women's Hospital, Helsinki University Central Hospital, where the EEG recordings were performed. The mothers or both parents of the infant gave an informed written consent to participation in the experiment. The study was approved by the Ethics Committee for Paediatrics, Adolescent Medicine and Psychiatry, Hospital District of Helsinki and Uusimaa.

Data from the infants who, according to the nurses' notes, were awake with eyes open for more than 1/6 of the time during the experiment were discarded from further analysis. Data from a total of 19 infants (male/female 11/8, 10 delivered with cesarean section) were accepted into the study. See Table 1 for detailed information on the participants.

**Table 1:** **Participant details (*n* = 19)**.

	**Age (d)**	**Duration of pregnancy (weeks + days)**	**Weight (g)**	**Height (cm)**	**5-min Apgar score**
Mean	1.7	39 + 6	3644	50	9.2
Range	1–4	37 + 6–42 + 3	2774–4260	45–54	9–10

### EEG experiment and stimuli

The oddball paradigm consisted of 551 stimuli (74% standards, 26% deviants, Table 2). The stimuli were triad chords comprised of three sinusoidal components (tones) with duration of 250 ms with 25 ms rise and fall times. They were presented to the participants in a pseudo-random order with a 1000-ms duration from the beginning of the stimulus to the beginning of next stimulus (stimulus-onset asynchrony, SOA) so that at least one standard stimulus preceded every deviant stimulus. The experimental paradigm is illustrated in Figure 1B.

**Table 2:** **The experimental stimuli (chords) and their note structure**.

**Standards**	**Chord name (notes)**	**%**	**Deviants**	**Chord name (notes)**	**%**
**Major triads**		73.9			26.1
	C-major (C′-E′-G′)	6.2	**Dissonant triads**		6.5
	D^b^-major (D^b′^-F′-A^b′^)	6.2		disson1 (E′-F′-B′)	2.2
	D-major (D′-F#′-A′)	6.2		disson2 (F#′-G′-C#″)	2.2
	E^b^-major (E^b′^-G′-B^b′^)	6.2		disson3 (G#′-A′-D#″)	2.2
	E-major (E′-G#′-B′)	6.2	**Minor triads**		6.5
	F-major (F′-A′-C″)	6.2		F-minor (F′-A^b′^-C″)	2.2
	F#-major (F#′-A#′-C#″)	6.2		F#-minor (F#′-A′-C#″)	2.2
	G-major (G′-B′-D″)	6.2		G-minor (G′-B^b′^-D″)	2.2
	A^b^-major (A^b′^-C″-E^b″^)	6.2	**Inverted major (2nd inversion)**		13.1
	A-major (A′-C#″-E″)	6.2		A-major (E′-A′-C#″)	4.4
	B^b^-major (B^b′^-D″-F″)	6.2		B^b^-major (F′-B^b′^-D″)	4.4
	B-major (B′-D#″-F#″)	6.2		B-major (F#′-B′-D#″)	4.4

*Notes are in two adjacent octaves, with lower octave marked with ′ and higher octave with ″*.

The standard stimuli were root major chords transposed to 12 frequency levels. Three deviant types were presented: root minor chords, inverted major chords (2nd inversion) and dissonant chords, each transposed to 3 frequency levels. The tones in the standard major chords ranged from the middle C (frequency 262 Hz) in C-major chord to F#5 (740 Hz) in B-major chord. The tones in the deviants were comprised of the same tones that were already present in the standard stimuli, and ranged from E4 (330 Hz) to D#5 (622 Hz). The stimuli were presented in three blocks of 9 min 11 sec. Deviants were presented a total of 108 times for the dissonant and minor chords, and 216 times for the inverted major chords.

During the recording, the infant was lying in a crib, with the head facing randomly either to the left or to the right, so that the other ear was partly obscured. The stimuli were presented from two loudspeakers placed outside the crib near the left and right corner close to the infant's feet. The sound level was about 60 dB SPL at the approximate location of the infant's head. The background noise level in the hospital room was approximately 46 dB SPL. The recording was performed by a trained nurse who observed the infant throughout the measurement, documenting the apparent sleep stages and activity of the infant. The infants in this study were in quiet sleep ~33% and in active sleep 67% of the time. Their hearing was normal according to the clinical routine screening with an otoacoustic emission test (EOAE, ILO88, Dpi, Otodynamics Ltd., Hatfield, UK).

### EEG recording and data quantification

EEG from 11 channels was recorded using Ag/Cl-electrodes placed on the infants' head according to the international 10/20-system (NeuroScan, Synamps 2 amplifier). Electrode locations F3, F4, C3, Cz, C4, P3, P4, T7, and T8 were used, with an additional electrode next to the infants right eye in order to monitor and record eye movements (electro-oculogram). The right mastoid was used as the reference. The sampling rate of the recording was 500 Hz.

The EEG was analyzed offline using BESA (v 5.3.7, BESA GmbH, Gräfelfing, Germany) analysis program. First, the EEG was filtered (high-pass 0.5 Hz, low-pass 30 Hz). Then it was divided into epochs of 600 ms, with a 100 ms pre-stimulus baseline, separately for each stimulus type and each participant on each electrode location. All epochs including voltage changes exceeding ±150 μV were omitted from further analysis in order to exclude artifacts.

In order to study the responses to deviants, difference waveforms were calculated by subtracting the standard ERP from the deviant ERP for each deviant type and each subject separately. For statistical analyses of standard and deviant responses, a time window was centered around the peak of the across-subjects mean waveform. Running-*t*-tests with 50 ms and 100 ms time windows were conducted for the standard wave and the deviant-minus-standard difference waves in order to explore the optimal latency window. The latency window chosen for the analyses was such that around it, latency windows starting from more than 10 consecutive time points gave a statistically significant *t*-test result on at least one electrode. This was done in order to minimize the risk of a false positive result in the *t*-tests for the responses. As a result, 100-ms windows 20–120 and 250–350 ms were used for the standard responses and a 50-ms window 240–290 ms for deviant-minus-standard responses.

Statistical analyses of standard and deviant-minus-standard responses were carried out for electrodes F3, F4, C3, C4, P3, and P4. One-sample *t*-tests were conducted for each stimulus type and each electrode in order to test the significance of the responses. The effect sizes of the *t*-tests were calculated with Matlab using Cohen's d (see Hentschke and Stüttgen, 2011). The scalp distribution of the standard and deviant-minus-standard responses was analyzed using repeated analysis of variance (ANOVA-R) with frontality (electrode lines F, C, P) and laterality (electrode lines left 3, right 4) dimensions. When sphericity could not be assumed, a Greenhouse–Geisser correction was used for the results. Statistically significant (*p* < 0.05) main effects or interactions were further tested with pairwise comparisons using Bonferroni correction.

## Results

### Response to standards

**Figure 2:**
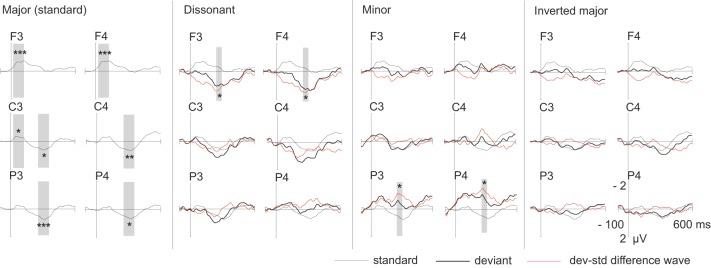
**Group-averaged (*n* = 19) ERP waveforms elicited by standard (thin line) and deviant stimuli (thick line) and the deviant-minus-standard subtraction waveform (red line).** The latency window used for statistical analyses is marked with the gray-shaded bars and statistical significance of the standard response or the deviant-minus-standard response is marked with ^*^'s (^*^p < 0.05, ^**^p < 0.01, ^***^p < 0.001).

The root major chords elicited an early negative response around 20–120 ms and a later positive response around 250–350 ms (Figure 2). The early negative response was statistically significant mostly on frontal electrodes and the late positive response on central and parietal electrodes (Table 3). ANOVA-R results showed that there was a statistically significant frontality effect for both the early negative response [*F*_(1, 305)_ = 12.991, *p* < 0.001] and the late positive response [*F*_(1, 249)_ = 5.703, *p* < 0.05]. However, the effect achieved statistical power only for the early negative response. In pairwise comparisons, the early negative response was larger on F-electrodes than on C- and P-electrodes (for both *p* < 0.01), and the late positive response was larger on C-electrodes than on F-electrodes (*p* < 0.01). ANOVA-R and pairwise comparison results for all standard and deviant responses are listed in Table 4.

**Table 3:** **The mean amplitude in μV (standard deviation) on the specified time window for the standard and deviant-minus-standard responses tested with one-sample *t*-tests**.

**Electrode**	**Mean (SD)**	***t* (dF)**	***p*-value**	**Cohen's d**	**Mean (SD)**	***t* (dF)**	***p*-value**	**Cohen's d**
	**Standard 20–120 ms**				**Standard 250–350 ms**			
**F3**	−0.61 (0.57)	−4.63 (18)	0.000^***^	1.49	−0.15 (1.06)	−0.61 (18)	0.552	0.17
**F4**	−0.61 (0.63)	−4.24 (18)	0.000^***^	1.37	−0.10 (1.11)	−0.39 (18)	0.704	0.10
**C3**	−0.29 (0.54)	−2.39 (18)	0.028^*^	0.76	0.54 (0.86)	2.73 (18)	0.014^*^	0.95
**C4**	−0.26 (0.64)	−1.77 (18)	0.094	0.55	0.58 (0.80)	3.15 (18)	0.006^**^	1.04
**P3**	−0.17 (0.42)	−1.74 (18)	0.099	0.53	0.65 (0.59)	4.79 (18)	0.000^***^	1.61
**P4**	−0.08 (0.64)	−0.56 (18)	0.583	0.15	0.58 (1.08)	2.33 (18)	0.032^*^	0.78
	**Dissonant 240–290 ms**				**Minor 240–290 ms**			
**F3**	1.29 (2.64)	2.13 (18)	0.047^*^	0.65	0.44 (2.94)	0.66 (18)	0.520	0.20
**F4**	1.46 (2.54)	2.51 (18)	0.022^*^	0.77	−0.17 (2.80)	−0.258 (18)	0.800	0.09
**C3**	0.61 (1.77)	1.51 (18)	0.149	0.41	0.02 (2.34)	−0.036 (18)	0.971	0.01
**C4**	0.68 (3.03)	0.98 (18)	0.341	0.33	−0.76 (2.99)	−1.11 (18)	0.284	0.38
**P3**	0.34 (1.81)	0.81 (18)	0.429	0.23	−1.02 (2.25)	−1.98 (18)	0.063	0.69
**P4**	−0.32 (2.16)	−0.66 (18)	0.521	0.19	−1.40 (2.70)	−2.26 (18)	0.036^*^	0.80

* p < 0.05;

** p < 0.01;

*** *p < 0.001*.

**Table 4:** **Results of the ANOVA-R showing *F*- and *p*-values for each response to laterality (left vs. right), frontality (frontal vs. central vs. parietal sites) and their interaction effects**.

**ANOVA-R**	**Effect**	***F* (df)**	***p*-value**	**Observed power**		**Pair-wise comparisons**	**Difference (SEM)**	***p*-value**
Standard 20–120 ms	Laterality	0.223 (1)	0.642	0.073	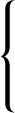	*F* vs. *C*	−0.332 (0.078)	0.001^**^
	Frontality	12.991 (1.305)	0.001^**^	0.966		*F* vs. *P*	−0.484 (0.128)	0.004^**^
	Interaction	0.214 (2)	0.808	0.081		*C* vs. *P*	−0.152 (0.077)	0.187
Standard 250–350 ms	Laterality	0.002 (1)	0.962	0.050	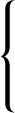	*F* vs. *C*	−0.682 (0.183)	0.005^**^
	Frontality	5.703 (1.249)	0.020^*^	0.687		*F* vs. *P*	−0.736 (0.323)	0.106
	Interaction	0.191 (2)	0.827	0.070		*C* vs. *P*	−0.054 (0.198)	1.00
Dissonant 240–290 ms	Laterality	0.228 (1)	0.639	0.074	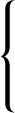	*F* vs. *C*	0.729 (0.495)	0.474
	Frontality	3.770 (2)	0.033^*^	0.651		*F* vs. *P*	1.369 (0.577)	0.087
	Interaction	0.897 (2)	0.417	0.192		*C* vs. *P*	0.640 (0.411)	0.411
Minor 240–290 ms	Laterality	2.720 (1)	0.116	0.345				
	Frontality	3.226 (1.329)	0.075	0.464				
	Interaction	0.339 (2)	0.715	0.100				

*Results of the pair-wise comparisons are shown for statistically significant ANOVA-R-effects*.

* p < 0.05;

** p < 0.01;

^***^ p < 0.001.

### Responses to deviants

*The dissonant chords* elicited a wide increased positive response most pronounced in the frontal electrodes (Figure 2, Table 3). ANOVA-R showed a statistically significant frontality effect [*F*_(2)_ = 3.770, *p* < 0.05]. However, the observed statistical power of the effect was low. In pairwise comparisons the response tended to be larger on F-electrodes than on P-electrodes (*p* = 0.087).

*The minor chords* elicited a negative response most visible on parietal electrodes (Figure 2, Table 3). In ANOVA-R, no effects of distribution (frontality/laterality) or interaction effects were found. ANOVA-R and pairwise comparison results for dissonant and minor chord responses are listed in Table 4.

*The inverted major chords* elicited no statistically significant responses (Figure 2), regardless of time window.

## Discussion

The main finding of our study was that newborn infants demonstrated a sensitivity to dissonant vs. consonant and major vs. minor chord categorizations, as evidenced by statistically significant MMRs to dissonant and minor chords in the context of consonant major chords. This occurred in the absence of deviant frequencies, with only the interval structure of the chords being the deviating factor between the standard and deviant chords. While dissonant chords elicited a frontal positive MMR in the context of major chords, minor chords elicited negative MMRs that were most pronounced over the parietal electrodes. This indicates that these categorizations were processed differently from each other by the infant brain. However, since the statistical power of the spatial distribution effects was low, these findings should be treated with caution. The infants' response to the standard major chords consisted of an early frontal negative response followed by a centro-parietal positive response. There was no significant MMR to the inverted major chords, contrasting with our hypothesis that inverted major vs. root major chords would be discriminated by newborn infants.

### Music sounds in the newborn brain

Our results of processing dissonant and minor chords support the previous findings that newborn infants are capable of abstract discriminations in the auditory domain (Ruusuvirta et al., 2003, 2004; Winkler et al., 2003; Carral et al., 2005). The current oddball paradigm with 12 different chords as standards and 9 chords as deviants, forming 3 groups of 3 chords, is even more complex than most of the paradigms previously used with infants, since in this paradigm the auditory system needs to extract the interval structure of the major chord, acting as standard, from the stimuli which are transposed to many different frequency levels. Also the SOA of 1 s is relatively long for infants, making it rather effortful to form and maintain the neural sound representations. In a previous study, presenting the stimuli too rapidly caused disappearance of MMR in infants (Cheour et al., 2002). The discrimination of the chord types with no deviant frequencies and only deviant interval structures requires processing of relational sound properties rather than absolute frequencies (in line with findings of, e.g., Tew et al., 2009).

In the present paradigm the deviant chords did not differ from standards in terms of frequency, i.e., all the frequencies in the deviants were already present in the standards. This was accomplished in order to ensure that the MMRs elicited by the deviants would be solely due to differences in the interval structure as opposed to involvement of new frequencies. Hence, our results demonstrate sensitivity to the interval structures of major vs. minor and consonant vs. dissonant chords in newborn infants. However, in both minor and highly dissonant chords, the lower interval size is smaller than in the root and inverted major chords. Thus, it can be argued that the MMRs elicited in the paradigm did not arise from differences in chord quality (major vs. minor, consonance vs. dissonance), but from differences in the lower interval size only. Unfortunately, separating chord quality from its interval structure altogether is impossible, since the chord quality is defined by the interval structure. However, especially dissonance can be created by various different interval structures but in the present paradigm, only one example of a dissonant interval structure was included. In future studies the paradigm could be further improved by using several types of chords instead of one chord transposed to several frequency levels to exemplify especially the dissonant chord quality. This would increase the likelihood that the MMR elicited by deviant chords would be due to their quality and not due to other properties of the chords.

Regarding the “musical infant,” our results support the view that consonance-dissonance discrimination is evident in the infants' hearing (e.g., Zentner and Kagan, 1998; Trainor et al., 2002; Perani et al., 2010). The major-minor result provides neural evidence extending the behavioral results of Crowder et al. (1991), suggesting that even though no preference might exist between major and minor in infancy, the major-minor-contrast is still automatically discriminated by the infants' auditory system in an oddball sequence. However, minor chords are slightly more dissonant than major chords, though still presently considered quite consonant in Western music. This raises the question whether major-minor discrimination in infants is more than just sensitivity to dissonance. An fMRI study of Western non-musician adults showed that dissonance only accounts for some of the processing differences of major vs. minor mode in the brain (Green et al., 2008). We suggest that this might be the case in the present study as well. The different polarity and spatial distribution of the responses to dissonant vs. minor chords support the view that the processing of the chord types differs more than just in terms of deviance magnitude.

The present results also extend the results from previous studies that have used a similar paradigm and stimuli in adults and school-aged children (Virtala et al., 2011, 2012). The newborn infants were capable of the same discriminations as non-musician adults (Virtala et al., 2011) and musically trained children (Virtala et al., 2012). However, it is noteworthy that minor chords elicited an MMR in the infants in the present study, while the non-musically trained children in our previous study did not show an MMN (Virtala et al., 2012). This difference in the automatic discrimination capabilities between infants and school-aged children might indicate the disappearance of an early sensitivity to major/minor distinction during development in the absence of musical training.

The inverted major chords, on the other hand, did not elicit MMRs in adults, children or infants in our studies. The difference between a chord and its inversion is perceptually small in Western music, since in many cases they can replace each other. However, when thinking about the absolute interval size, the difference between a chord and its inversion is larger than the difference between root major and root minor chords. While major and minor chords are separated by a one-semitone difference in one of their notes, a root chord and its inversion are separated by a whole-octave difference in one note. Hence, it is possible that to someone unfamiliar with Western music, the inverted major chord and root major chord would sound different in the same way as major and minor chord. Several studies have shown evidence of a more culture-independent processing of music in infants than in adults (see, e.g., Trainor and Trehub, 1992). However, compared to the minor chord, both root and inverted major chords are more consonant, since there are less dissonant combinations in their harmonic partials. Hence, the degree of dissonance in the chord types might solely explain why the inverted major chords were not discriminated from root major chords in this study. Furthermore, in this study, the chords were comprised of sinusoidal tones and thus did not include harmonics. This might affect the processing differences of the chord types by making the differences between root major, inverted major and root minor chords smaller than in chords with harmonics.

The consonance-dissonance dichotomy might be culture-independent in humans and present in some other species as well (see, e.g., Hulse et al., 1995; Fritz et al., 2009). The result that infants are sensitive to this dichotomy could thus be interpreted as a biological predisposition of musical skills at birth. Hannon and Trainor (2007) have suggested that the early sensitivity to dissonance might be a universal building block in human hearing. Whether the difference between major vs. minor modes should be considered a cultural construction or a somewhat “innate” dichotomy is unclear, and further empirical work in future is needed. Our results, however, introduce pioneering findings of sensitivity to major vs. minor in the newborn brain.

### Developing ERPs in infancy

Earlier studies have demonstrated that infant MMR's may vary in polarity (Trainor et al., 2003; He et al., 2007, 2009), which could reflect differences in maturation (Porges et al., 1999; Leppänen et al., 2004). According to the present results, both the positive and the negative auditory MMRs in infants are real phenomena and, in our view, *parallel rather than mutually exclusive*. Since positive and negative MMRs were elicited in the same measurement, a purely maturational account cannot fully explain the current results (Porges et al., 1999; Leppänen et al., 2004). If positive and negative MMRs occur at the same time, they may have a different cortical origin (as suggested by He et al., 2007; Trainor, 2012). For example, an earlier study using speech sounds reported both positive and negative MMRs in infants—the positivity was most often observed when the infant was asleep but the negativity *only* when the infant was awake (Friederici et al., 2002). Since the positive MMR was present both during sleep and wakefulness, it was interpreted to reflect precognitive processing of auditory information. Furthermore, since the negative MMRs were only present while awake and hence seemed to call for alertness, it was hypothesized that they might reflect a more cognitive process. In other words, the authors suggested that two change-related responses might exist in parallel, reflecting different underlying cognitive and neural functions in the developing brain.

Infants' ERP responses to standard stimuli in oddball experiments have been small and negative in some studies (see, e.g., Leppänen et al., 1997; Ruusuvirta et al., 2003, 2004) and positive in others (see, e.g., Cheour et al., 2002; Trainor et al., 2003). In general, auditory ERP components other than the MMN have been considered very immature in the infants' brain (see, e.g., Kushnerenko et al., 2002a,b). In this study we found statistically significant responses to standards: an early frontal negativity followed by a later centro-parietal positivity. More research is needed to examine, how these results correspond to the ERPs reported in, e.g., young children (Kushnerenko et al., 2002a,b).

To conclude, our study introduces a complex oddball paradigm with several deviant types and varying standards in the ERP studies of newborn infants. We found that both dissonant vs. consonant and minor vs. major chord discriminations take place at the pre-attentive level of central auditory processing in newborns. The neural processing of these two categorizations seems to differ, since the minor chords elicited a negative change-related response and the dissonant chords a positive enhancement in the context of consonant major chords. Our results show evidence of abstract categorizations in infant auditory processing. Furthermore, consonance-dissonance and major-minor dichotomies seem to be meaningful already in infants' hearing.

### Conflict of interest statement

The authors declare that the research was conducted in the absence of any commercial or financial relationships that could be construed as a potential conflict of interest.
